# Uncontrolled pain in critically ill patients and acute kidney injury: a hypothesis-generating cohort study

**DOI:** 10.1186/s12882-022-02810-x

**Published:** 2022-06-03

**Authors:** Laura Herranz Prinz, Isac de Castro, Luciano de Cesar Pontes Azevedo, Jose Mauro Vieira

**Affiliations:** Research and Education Institute, Hospital Sírio Libanês, 69, Daher Cutait – Bela Vista – São Paulo (SP), São Paulo, CEP 01308-060 Brazil

**Keywords:** Acute pain, Acute kidney injury, Inflammation, Organ crosstalk, Critical care

## Abstract

**Background:**

In critically ill patients, acute pain occurs frequently, causes sympathetic activation, release of inflammatory mediators, and potential organ dysfunction, with the kidneys potentially sensitive to inflammation-mediated injury. This study aimed to explore the association between acute pain in critically ill patients and the occurrence of acute kidney injury (AKI).

**Methods:**

Data from a retrospective cohort of adult patients admitted between June 2013 and June 2016 to the Intensive Care Unit (ICU) of a tertiary hospital in São Paulo, Brazil, were analyzed. The main exclusion criteria were ICU length of stay < 48 h, coma, and prior kidney dysfunction. The outcome (AKI) was defined as an elevation in the baseline serum creatinine level of ≥ 0.3 mg/dl and/or > 50% at any time after the first 48 h in the ICU. Multivariable logistic regression and hierarchical cluster analysis were performed.

**Results:**

The isolated incidence of pain was 23.6%, and the incidence of pain duration > 5 days was 10.6%. AKI occurred in 31.7% of the cohort. In multivariable logistic analysis, duration of pain > 5 days (OR 5.25 CI 2.19–12.57 *p* < 0.01) and mechanical ventilation (MV) ≥ 3 days (OR 5.5 CI 2.3–13.5 *p* < 0.01) were the variables with positive association with AKI. The hierarchical cluster analysis reinforced the relation between AKI, MV and duration of pain.

**Conclusions:**

Pain is an especially important issue in critically ill patients and in this exploratory study it appears to be associated with AKI development. The search for more rigorous pain control in ICU is crucial and can influence organ dysfunction.

**Graphical Abstract:**

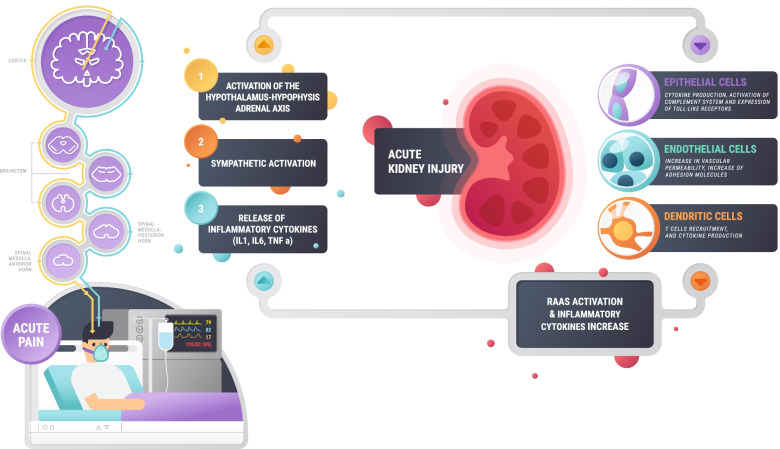

## Background

Pain is the most common symptom among critically ill patients in intensive care unit (ICU), and it is poorly controlled in most ICUs throughout the world [1–4]. The ICU is an environment in which multiple painful interventions and procedures take place [2], but despite the growing importance of this subject [5], the prevalence of pain remains significant, especially among seriously ill patients [2, 6].

The literature emphasizes the evidence linking suboptimal pain control to poorer clinical outcomes. Acute pain apparently cause delirium [7] and prolonged mechanical ventilation (MV) [8]. In observational studies, those whose pain was treated properly required lower doses of hypnotics and benzodiazepines, resulting in less time on MV and shorter hospital length of stay (LOS), as well as a lower risk of ventilator-associated pneumonia (VAP) [8, 9]. In surgical patients, proper pain control reduced incidence of cardiovascular events and pulmonary thromboembolism [10, 11]. The use of epidural anesthesia/analgesia in high-risk surgical patients can reduce perioperative surgical stress, through sympathetic modulation and the reduction of inflammatory cytokines, with a consequent reduction in myocardial oxygen consumption and improvement in overall organ perfusion [12].

Although it is known that pain can cause metabolic stress, autonomic activation, and the activation of inflammatory cells [13–15], it remains little explored how poor pain control can have direct consequences for several distant organs. Recently, there has been increased knowledge of the bidirectional impacts of distant dysfunctional systems or organs; a phenomenon also referred to as organ crosstalk [16]. Pioneering studies have been published on acute kidney injury (AKI) and its effects on other organs, such as the lungs [17]. The kidneys are examples of organs especially sensitive to injury mediated by systemic effects of metabolic stress and inflammatory response [18–20].

Thus, we hypothesized that a painful state might be conceivably associated to distant organ signaling, resulting in predisposition to AKI. Thus, the objective of this study was to explore the relationship between acute pain and AKI in a cohort of critically ill patients.

## Methods

### Study design and database

This study was conducted with a retrospective cohort of adult patients admitted to the ICU of Hospital Sírio Libanês, a 480-bed tertiary hospital in São Paulo, Brazil, between 1 June 2013 and 30 June 2016. All the data in the current study were extracted from an integrated electronic medical records of Hospital Sírio Libanês (Epimed® and Philips Tasy® Platforms), with approval of the Ethics Committee of the Institute for Education and Research at Hospital Sírio Libanês (access number 445). All the patients in the database were de-identified for privacy protection, and the need for informed consent was waived because of its observational nature.

### Inclusion and exclusion criteria

The study included all adult patients aged > 18 years who were admitted to the general, mixed, 30-beds ICU during the study period and remained there for at least 48 h. The exclusion criteria were any baseline creatinine elevation within the first 48 h of admission, chronic kidney dysfunction comorbidity (with or without dialysis), transplanted kidney, coma or delirium, or intracranial tumor associated with a diagnosis of intracranial hypertension at admission; admission due to cardiorespiratory arrest; palliative care mandate; and readmission within 24 h.

### Data extraction

For each patient, we collected information on demographic characteristics, diagnosis, cause of ICU admission, comorbidities, physiological and laboratory findings [i.e., C-Reactive Protein (CRP)], organ support during ICU stay [i.e., renal replacement therapy (RRT), mechanical ventilation (MV), use of vasoactive drugs and sedatives], and severity scores for organ dysfunction [i.e., Simplified Acute Physiology Score 3 (SAPS 3) and Sequential Organ Failure Assessment (SOFA)] from the first day of hospitalization until the outcome (AKI). The SOFA score data was analysed in first 24 h and stratified according to dysfunctions.

Pain was evaluated using electronic registry data recorded every two hours by the care teams. Patients who could communicate self-rated their pain using a Numerical Verbal Scale (NVS; 0–10), and those who could not communicate and/or were on mechanical ventilation were evaluated using a categorical Behavioral Pain Scale (BPS; yes/no for any of these events: facial expression, unusual upper limbs movements and compliance with ventilator, when appropriate). We also examined the duration (persistence) of pain with consideration of NVS scores in the presence of any value other than 0 and categorical consideration of BPS scores. Each patient’s need for analgesia (non opioids – principally metamizole—and strong opioids—morphine, methadone and fentanyl) was also evaluated through the grouping of analgesic dosages during ICU stay.

The variables of interest had continuous data categorized using the Receiver Operating Characteristic (ROC) curve to determine the best accuracy against the clinical outcome (AKI), with the cutoff score for each variable determined by the highest value of the sum between sensitivity and specificity, corresponding to the point of greatest inflection of the ROC curve, with the following predictors being determined: age > 70 years, MV ≥ 3 days, NVS ≥ 3 or positive pain in BPS, pain duration > 5 days, CRP ≥ 5.2 mg/dl, SOFA ≥ 4 points, SAPS3 ≥ 48 points and absence or presence of use of nephrotoxic drugs, sedatives, vasoactive drugs and use of non-opioid analgesics and strong opioids (yes/no).

### Outcome definition

The primary outcome was AKI (according to adapted AKIN criteria), defined as a ≥ 0.3-mg/dl and/or > 50% increase in the creatinine level only after the first 48 h of ICU stay (in relation to the patient’s creatinine level at admission). The creatinine level after the first 48 h of ICU was considered to make the timeframe association between the independent predictors and AKI plausible.

### Statistical analysis

Continuous variables are expressed as mean ± standard deviation or median (interquantile range), as appropriate. Continuous and semi-continuous data were compared with the Gauss curve and determined to be non-parametric by the Kolmogorov–Smirnov test. Given the non-parametric nature of continuous data for the total sample, we compared them using the Mann–Whitney test. Categorical and categorized data were represented as absolute and relative frequencies and analyzed using Pearson’s chi-squared test with bootstrap adjustment for large samples. We then performed multiple (binary) logistic regression, with the variables of minor influence being removed one by one (“step by step”), until the model presents cohesion (*p* < 0,05). Then, the division of the cohort was carried out into two groups according to the odds ratios (ORs; *p* < 0.05) in the bivariate analysis, forming groups with ORs of < 2.5 [compartment 1 (C1)] and ≥ 2.5 [compartment 2 (C2)]. This OR cutoff value was determined by the ROC curve, and the compartmental model was applied for major performance of the logistic regression, with the variables grouped by similarity. Compartment 1 (C1) included the variables age, gender, stratified SOFA (cardiovascular, hepatic, hematological), comorbidities (cirrhosis, dementia, hematological tumors) and use of strong opioids and non-opioid analgesics. Compartment 2 (C2) included in the analysis MV, global and stratified SOFA (neurological and respiratory), CRP-t ≥ 5.2 mg/dl, use of sedatives, nephrotoxic drugs, vasopressors and SAPS3. Both compartments were analysed in multiple logistic regression in association with the predictors of pain: NVS ≥ 3 or positive pain by BPS and pain duration > 5 days.

The hierarchical cluster analysis was carried out to determine the association of the study variables in relation to the outcome, according to similarity and hierarchical representation of them [dendrogram 1 (D1) and dendrogram 2 (D2)]. The variables were organized by average linkage within groups to elements of a tree, where the leaves represent the variables of patients and the length between nodes represent the distance between them determined by Pearson correlation test, and similar variables are found in the same sub-trees.

In this study, an *α* ≤ 5% risk for type I error and a *β* ≤ 20% risk for type II error were considered to be acceptable. Data were analyzed using IBM SPSS® Statistics, version 23.0 (Armonk, NY: IBM Corp) and GraphPad Prism, version 5.00 for Windows (San Diego, CA).

## Results

### Baseline characteristics

**Fig. 1:**
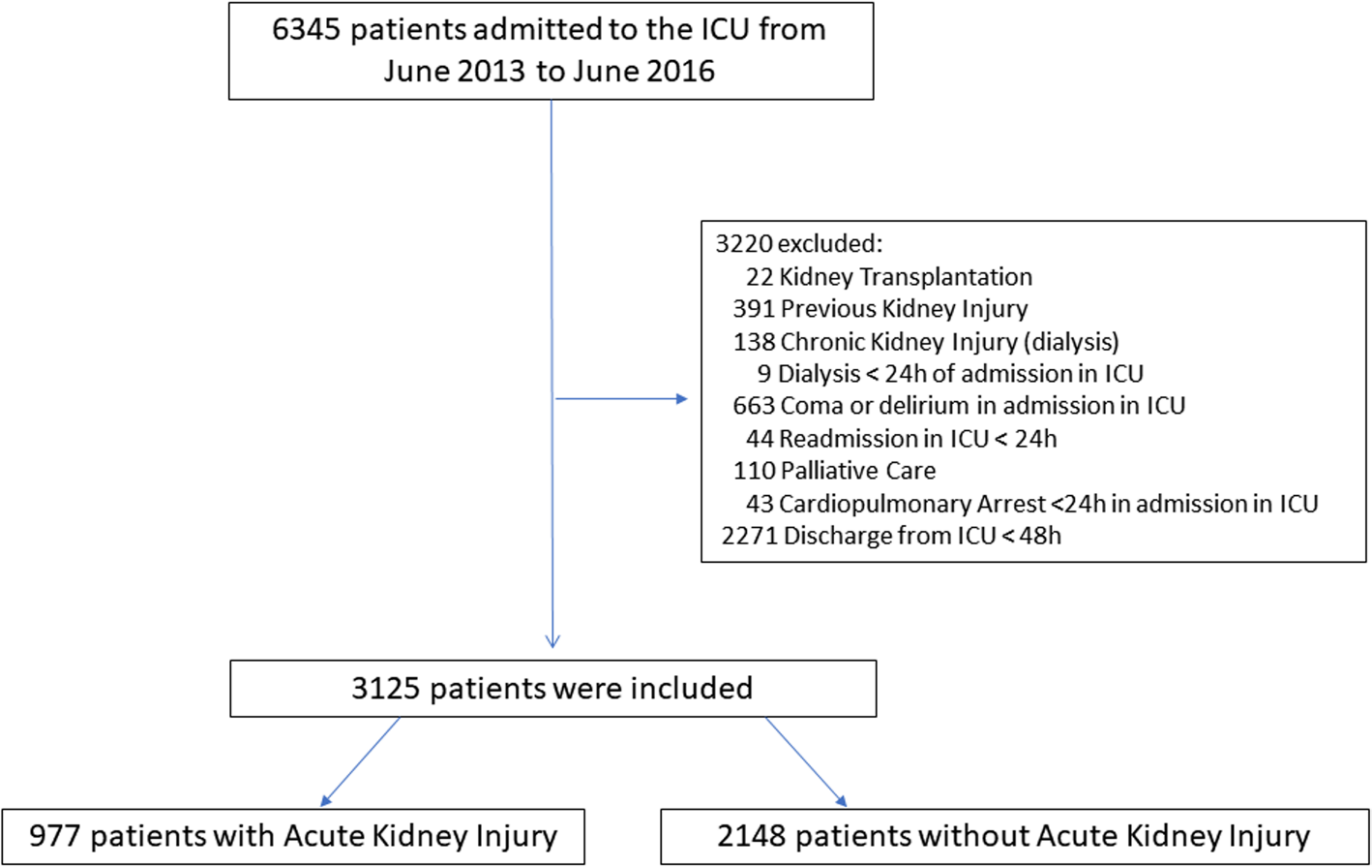
Flowchart of study patients. Legend: ICU – Intensive Care Unit

Data from 3125 patients admitted to the ICU during the study period were included. Figure 1 illustrates the flow chart of patient selection. Table 1 shows the demographic and clinical characteristics of the included patients.

**Table 1:** Baseline characteristics of patients in the cohort

Demographic data	All patients (*n* = 3125)
Age (years), median (IQR)	**69 (55–81)**
Male, n (%)	**1595 (51.0)**
Admission type, n (%)	
Emergency surgery	**246 (7.9)**
Elective surgery	**1104 (35.3)**
Clinical	**1585 (50.7)**
Admission diagnostic, n (%)	
Cardiovascular	**178 (5.7)**
Respiratory	**215 (6.9)**
Liver	**261 (8.4)**
Gastrointestinal	**147 (4.7)**
Neurologic	**216 (6.9)**
Sepsis	**658 (21.1)**
Gastrointestinal surgery	**315 (10.1)**
Neurosurgery	**170 (5.4)**
Comorbidities, n (%)	
NYHA	**151 (4.8)**
Cirrhosis	**53 (1.7)**
Solid tumors	**1216 (38.9)**
Hematologic tumors	**153 (5.2)**
Arterial Hypertension	**1172 (53.7)**
Diabetes mellitus	**623 (19.9)**
Dementia	**219 (10)**
Disease severity score (points), median	
SOFA score on ICU admission (24 h)	**2 (1–4)**
SAPS3 score on ICU admission (1 h)	**40 (32–50)**
Mechanical Ventilation, n (%)	**509 (16.2)**
Vasopressors, n (%)	**906 (28.9)**
Pain, n (%)	
NVS ≥ 3 or BPS positive	**739 (23.6)**
Pain duration > 5 days	**333 (10.6)**
Non opioid use	**874 (27.9)**
Strong opioids use	**509 (16.2)**
Acute Kidney Injury – Outcome, n (%)	**977 (31.7)**
Hospital mortality, n (%)	**332 (10.6)**

*NYHA* – New York Heart Association; *SOFA* – Sequencial Organ Failure Assessement; *SAPS*—Simplified Acute Physiology Score; *NVS* – Numerical Verbal Scale; *BPS*—Pain Behavioural Scale

Of this cohort, 47% (*n* = 1470) of patients were aged > 70 years, 9.1% (*n* = 286) remained on MV for ≥ 3 days, and 32% (*n* = 1000) of patients had SAPS 3 scores ≥ 48 points. The in-hospital mortality rate was 10%. The incidence of NVS score ≥ 3 or positive BPS finding was 23.6% (*n* = 739). The incidence of pain duration > 5 days was 10.6% (*n* = 333). The need for non-opioids analgesics was observed in 27.9% of patients, and strong opioids were used in 16.2% of cases. Elevated levels of C-Reactive Protein (CRP) (≥ 5.2 mg/dl) were found in 72.2% of the cohort. Evidence of AKI development > 48 h after ICU admission was observed in 977 (31.7%) patients (Table 1).

### Bivariate findings

In the bivariate analysis, the use of MV was associated with AKI [OR 6.3, 95% confidence interval (CI) 4.4–9.1; *p* < 0.05; Table 2] as well as the duration of pain (> 5 days), with an OR of 12.05 (95% CI 8.6–16.8). The presence of pain (NVS ≥ 3 or BPS positive), use of non-opioids analgesics, and use of strong opioids were related to AKI, with ORs of 1.5 (95% CI 1.3–1.9), 1.7 (95% CI 1.4–2.0), and 2.2 (95% CI 1.8–2.7), respectively. Serum CRP values ≥ 5.2 mg/dl were also associated with AKI (OR 4.6, 95% CI 3.7–5.9; *p* < 0.05; Table 2).

**Table 2:** Bivariate analysis between independent variables and Acute Kidney Injury (AKI), including pain

Variables	No AKI (*n* = 2148)	AKI (*n* = 977)	OR (IC 95%)	*P* value
Age, n (%)				
> 70 years	**960 (4.6)**	**510 (44.6)**	**1.3 (1.1–1.5)**	** < 0.05**
Male, n (%)	**1021 (47.5)**	**551 (56.3)**	**1.3 (1.1–1.5)**	** < 0.05**
Comorbidities, n (%)				
NYHA	**95 (4.4)**	**56 (5.7)**	**1.2 (0.9–1.8)**	**0.145**
Cirrhosis	**29 (1.3)**	**24 (2.4)**	**1.8 (1.0–3.1)**	** < 0.05**
Solid tumors	**837 (38.9)**	**363 (37.1)**	**0.8 (0.7–1.0)**	**0.164**
Hematologic tumors	**84 (3.9)**	**68 (6.9)**	**1.8 (1.3–2.5)**	** < 0.05**
Arterial Hypertension	**783 (36.4)**	**372 (38)**	**1.0 (0.8–1.2)**	**0.469**
Diabetes mellitus	**413 (19.2)**	**204 (20.8)**	**1.0 (0.8 -1.3)**	**0.419**
Dementia	**130 (6.0)**	**86 (8.8)**	**1.5 (1.1–2.0)**	** < 0.05**
SOFA (daily), n (%)				
≥ 4 points	**308 (14.3)**	**395 (40.4)**	**5.1 (4.2–6.2)**	** < 0.05**
SOFA on ICU admission—stratified ≥ 1 point, n (%)				
Neurologic	**379 (17.6)**	**324 (33.1)**	**2.6 (2.1–3.1)**	** < 0.05**
Respiratory	**290 (13.5)**	**269 (27.5)**	**2.6 (2.2–3.2)**	** < 0.05**
Cardiovascular	**1006 (46.8)**	**571 (58.4)**	**2.2 (1.8–2.8)**	** < 0.05**
Hematologic	**581 (27.0)**	**359 (36.7)**	**1.7 (1.4–2.0)**	** < 0.05**
Hepatic	**137 (6.3)**	**132 (13.5)**	**2.3 (1.8–3.0)**	** < 0.05**
SAPS3 on ICU admission (1 h), n (%)				
≥ 48 points	**539 (25)**	**465 (47.5)**	**2.6 (2.2–3.0)**	** < 0.05**
Mechanical Ventilation, n (%)				
≥ 3 days	**58 (2.7)**	**228 (23.3)**	**6.3 (4.4–9.1)**	** < 0.05**
Vasopressors use, n (%)	**494 (22.9)**	**412 (42.1)**	**2.5 (2.1–2.9)**	** < 0.05**
Pain, n (%)				
NVS ≥ 3 or BPS positive	**435 (20.2)**	**304 (31.1)**	**1.5 (1.3–1.9)**	** < 0.05**
Pain duration > 5 days	**92 (4.2)**	**241 (24.6)**	**12.0 (8.6–16.8)**	** < 0.05**
Non opioid use	**518 (24.1)**	**356 (36.4)**	**1.7 (1.4–2.0)**	** < 0.05**
Strong opioids use	**265 (12.3)**	**244 (24.9)**	**2.2 (1.8–2.7)**	** < 0.05**
CRP, n (%)				
≥ 5,2 mg/dl	**1374 (63.9)**	**883 (90.3)**	**4.6 (3.7–5.9)**	** < 0.05**
Sedative use, n (%)	**44 (2.0)**	**107 (10.9)**	**5.6 (3.9–8.0)**	** < 0.05**
Nephrotoxic drug use, n (%)	**36 (1.6)**	**95 (9.7)**	**6.0 (4.0–8.9)**	** < 0.05**

*AKI* – Acute Kidney Injury; *NYHA* – New York Heart Association; *SOFA* – Sequencial Organ Failure Assessement; *SAPS*—Simplified Acute Physiology Score; *NVS* – Numerical Verbal Scale; *BPS*—Pain Behavioural Scale; *CRP* – C-Reactive Protein

### Multivariate findings and clustering analysis

**Fig. 2:**
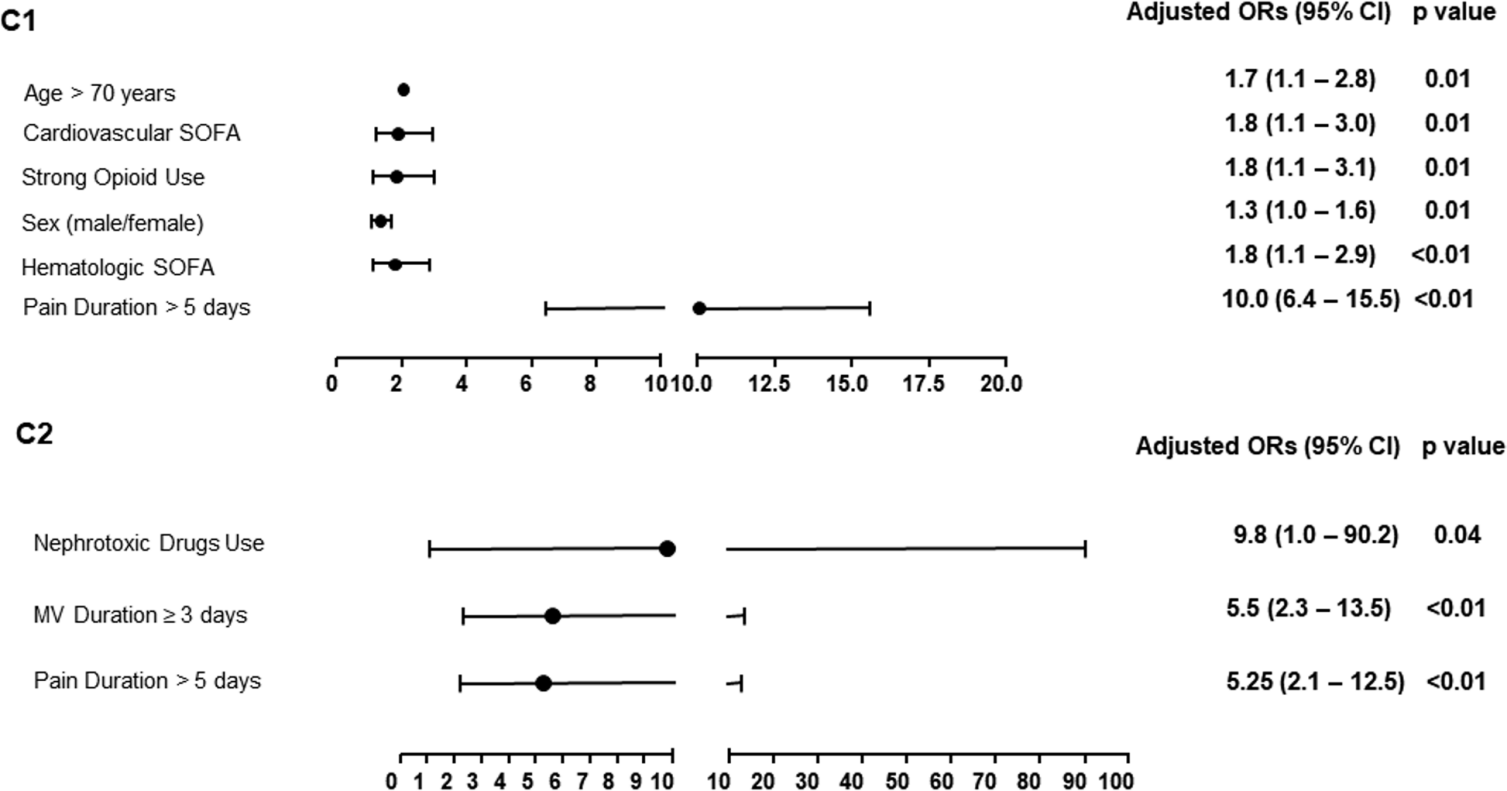
Regression logistic analysis of the association between pain and other risk factors for acute kidney injury in critical ill. Legend: SOFA – Sequencial Organ Failure Assessement; MV – Mechanical Ventilation

In the binary logistic regression model for compartment 1 (C1), AKI was associated independently with sex and age [ORs, 1.3 (95% CI 1.0–1.6) and 1.7 (95% CI 1.1–2.8), respectively], stratified cardiovascular and hematological SOFA scores [OR 1.8 (95% CI 1.1–3.0) and OR 1.8 (95% CI 1.1–2.9), respectively], pain duration > 5 days and use of strong opioids [OR 10.0 (95% CI 6.4–15.5) and OR 1.8 (95% CI 1.1–3.1), respectively; all *p* ≤ 0.01; Fig. 2]*.* For compartment 2 (C2), AKI was associated with pain duration > 5 days (OR 5.25, 95% CI 2.19–12.57), the use of MV for ≥ 3 days (OR 5.5, 95% CI 2.3–13.5; both *p* < 0.01), and with the use of nephrotoxic drugs (OR 9.8, 95% CI 1.0–90.2; *p* = 0.04; Fig. 2).

**Fig. 3:**
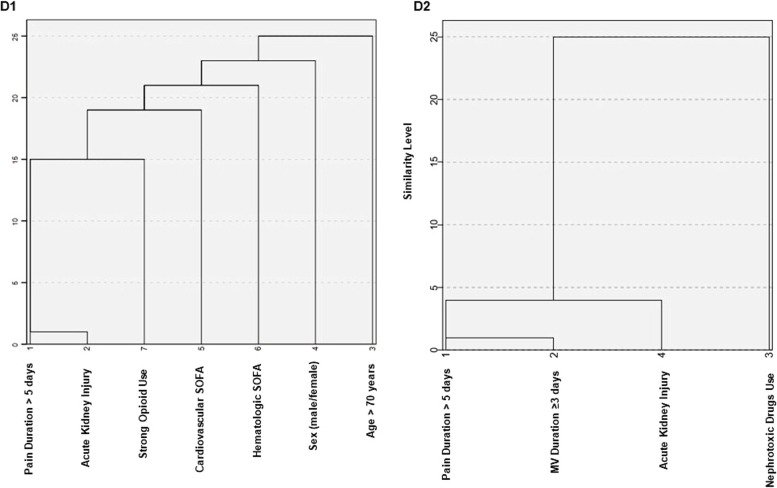
Hierarchical cluster association between pain duration, acute kidney injury and the other risk factors. Legend: SOFA – Sequencial Organ Failure Assessement; MV – Mechanical Ventilation

In the dendrogram 1 (D1) generated by hierarchical clustering, AKI showed the greatest similarity (clustered) with the duration of pain (Fig. 3). At a similarity level of about 15, the use of strong opioids and the duration of pain clustered with the outcome. In the dendrogram 2 (D2), AKI clustered with the duration of pain and MV at a similarity level of about 4 (Fig. 3).

## Discussion

In the cohort studied, AKI appeared to be associated with pain and its duration, corroborating the likelihood of pathological downstream systemic effects of pain experience in target organs.

The 26.6% incidence of pain in our sample is slightly lower than that reported in the literature for hospitalized patients [3, 21], possibly due to the regular use of analgosedation in clinical and preemptive/multimodal analgesia in surgical patients. In a mixed ICU study (i.e., including patients with and without mechanical ventilation, as in the present study), the incidence of pain was 51%; clinical and surgical patients were found to have elevated incidences of pain, perhaps attributable to the high levels of sepsis/inflammation and low historical application of preemptive analgesia [4].

As the literature overly shows, pain needs to be assessed repeatedly every day, and no single scale is enough for the correct interpretation of pain. In this study, we used the numerical verbal scale (NVS) for patients who were able to report their pain and the BPS for non-communicative patients; both scales are recommended (and validated) for clinical and surgical patients [22], and have been widely used [4, 9, 23]. In a study of interobserver reliability (among the attending nurse, researcher, and patients themselves, when possible), the NVS and BPS showed good reliability (*k* = 0.71 and *k* = 0.67, respectively) [24]. Newer measurement methods are not yet widely used and calibrated for the ICU [25, 26].

The intensity and duration of pain were associated with AKI in this study; these associations have not been explored thoroughly in previous studies. We used a cutoff value of NVS score > 3 when estimating pain intensity in communicative patients, similar to cutoff values reported in the literature [9]. With respect to the evaluation of pain intensity in non-communicative patients, behavioral scales are known to have qualitative restrictions, as the data are binary and categorical (i.e., yes, or no) [27], making comparisons between intensities of the various studies most difficult. In this study, pain duration > 5 days were associated with AKI in the multivariate and hierarchical clustering analyses. Desbiens et al. [28] reported that critically ill patients with high levels of pain (in terms of frequency and intensity) during hospitalization had higher incidences of (persistent) pain 2–6 months after discharge. Peters et al. [29] reported that critical surgical patients with high levels of pain after the fourth postoperative day were at high risk of (chronic) pain, functional limitations, and compromise of quality of life during 6 months of follow-up. Another study evaluating the trajectory of pain over the first 6 postoperative days showed that 60% of patients had at least moderate pain after the fourth day of hospitalization, and 12% showed an increase in pain during this period, suggesting an association with poorer outcomes [30]. A similar study of the trajectory of pain in the hospital on postoperative days 2–6 with readmission within 30 days showed that patients with ascending pain trajectories (55% of the cohort) had higher levels of complications, making them more susceptible to readmission [31]. Poorly controlled pain may be associated with an insufficient period of analgesia, low doses of analgesics because of their side effects, prior chronic pain, the type and/or duration of exposure to painful stimuli, and/or insufficient central blocking associated with the inadequate use of analgesics. Approximately 10% of patients in the cohort had persistent pain and were more susceptible to poorer outcomes related to pain, including AKI, posttraumatic stress, and chronic pain, as described [32]. There is notwithstanding a gap in the literature regarding the relationships between the intensity and duration of pain and poorer clinical outcomes.

The use of strong opioids was associated with AKI in the multivariate analysis most likely because of it´s a robust surrogate marker of intense pain. Although the use of opioids is the standard option for the control of pain symptoms in critically ill patients and occurred in nearly one-fifth of cases in this study, the many side effects of these drugs, are well known [33]. In our study only an insignificant patients of cohort received non-steroidal anti-inflammatory drugs (NSAIDs), and this surrogate for pain could not be account for AKI.

We hypothesize that the downstream effects of the painful state, well acknowledged regarding myocardial infarction risk and thromboembolic complications could as well lead to AKI risk, by the conceivable means of systemic inflammation, sympathic-mediated renal vasoconstriction, just to mention a few potential physiopathological mechanism. Recently, the sedation and pain management in ICU and surgical patients has been received attention, as there might be renal protective profiles based on the use of different treatment approaches [34–36].

Likewise, there are hitherto unanswered questions: which objective measure of pain in patients who are unable to communicate is best [25]; which serum biomarkers for pain might help with this diagnosis [37]; which combinations and dosages of analgesic drugs alongside opioids are more adequate [38]; and what other negative outcomes in distant organs acute pain might trigger or contribute to. Considering the ethical difficulties encountered in clinical studies of acute pain the ICU, our study included a large number of patients, with a mixed group of surgical and clinical indication, representative of the real situation in most ICUs and permitting relational inference based on a retrospective cohort.

Nevertheless, this study has certain limitations. Due to its retrospective design, it is subject to information bias and confounding factors; the precision and completeness of data are relevant issues in studies of this type, although few data were missing for our sample. The study was also conducted in a single ICU, which could have influenced the composition of the sample, and it involved the application of local therapeutic strategies. Furthermore, the global incidence of pain may have been underestimated because of the current regular use of common analgesics and drugs with analgesic potential (the analgosedation strategy), such as dexmedetomidine, in the postoperative period and for patients on MV, and because of the presence at the hospital of a specialized pain control group, which was represented well in discussions about analgesia and analgesic protocols. In the postoperative period, many patients depend on multimodal and/or patient-controlled analgesia, which was not examined in this study. We also did not analyze the use of other, less common therapies (e.g., non-pharmacological) applied to critically ill patients, or the incidence of prior chronic pain because of their low levels of representation. In summary, this study provides preliminary data for hypothesis generation of a relationship between the pain and AKI. Whether it is pain or some combination that might be associated with changes in kidney function cannot be completely discerned. Therefore, further studies and more robust analytical studies are strongly encouraged.

## Data Availability

Available through correspondence with the authors. Data were extracted from an integrated electronic medical records of Hospital Sírio Libanês (Epimed® and Philips Tasy® Platforms) and analyzed using IBM SPSS® Statistics and GraphPad Prism.
